# Acknowledgement of manuscript reviewers 2015

**DOI:** 10.1186/s12970-016-0116-0

**Published:** 2016-02-17

**Authors:** Jose Antonio, Douglas Kalman, Richard Kreider

**Affiliations:** NOVA Southeastern University, Fort Lauderdale, USA; Miami Research Associates, Miami, USA; Texas A&M University, College Station, USA

## Abstract

The editors of *Journal of the International Society of Sports Nutrition* would like to thank all our reviewers who have contributed to the journal from Volume 12 (2015).

**Adriana Coletta**

United States of America

**Alan Aragon**

United States of America

**Alan Walker**

United States of America

**Alexei Wong**

United States of America

**Andrew Barniger**

United States of America

**Andrew Jagim**

United States of America

**Andrew Jones**

United Kingdom

**Andrew Shao**

United States of America

**Anthony Almada**

United States of America

**Anya Ellerbroek**

United States of America

**Beat Knechtle**

Switzerland

**Benjamin Carr**

United States of America

**Benjamin Wax**

United States of America

**Bill Campbell**

United States of America

**Brad Schoenfeld**

United States of America

**Bruno Gualano**

Brazil

**Bryan Chung**

United States of America

**Brynmor Breese**

United Kingdom

**Catalina Casaru**

United States of America

**Chad Kerksick**

United States of America

**Chase Hollmer**

United States of America

**Chris Harnish**

United States of America

**Chris Lockwood**

United States of America

**Christopher Mobley**

United States of America

**Christopher Scott**

United States of America

**Colin Wilborn**

United States of America

**Conrad Earnest**

United States of America

**Dan Benardot**

United States of America

**Daniel Jaffe**

United States of America

**Darren Candow**

Canada

**Darryn Willoughby**

United States of America

**David Bellar**

United States of America

**Declan Connolly**

United States of America

**Douglas Kalman**

United States of America

**Edward Weiss**

United States of America

**Elfego Galvan**

United States of America

**Emma Stevenson**

United Kingdom

**Eric Trexler**

United States of America

**Erico Caperuto**

Brazil

**Eva Blomstrand**

Sweden

**Fernando Naclerio**

United Kingdom

**Gaetano Cairo**

Italy

**Geoffrey Hudson**

United States of America

**Glyn Howatson**

United Kingdom

**Hector Lopez**

United States of America

**Hubert Lee**

United States of America

**James Fell**

Australia

**Jason Cholewa**

United States of America

**Jean Farup**

Denmark

**Jeffrey Stout**

United States of America

**Jenna Becker**

United States of America

**Jennifer Bunn**

United States of America

**Jeremy Loenneke**

United States of America

**Jernej Kapus**

Slovenia

**Joan Eckerson**

United States of America

**Joel Cramer**

United States of America

**Jonathan Oliver**

United States of America

**Jordan Joy**

United States of America

**Jose Antonio**

United States of America

**Joseph Pellegrino**

United States of America

**Juan del Coso Garrigós**

Spain

**Julie Minevich**

United States of America

**Kamal Patel**

Canada

**Krista Austin**

United States of America

**Kyle Levers**

United States of America

**Laurent Bannock**

United Kingdom

**Leigh Breen**

United Kingdom

**Lemuel Taylor**

United States of America

**Mark Fogarty**

United Kingdom

**Mark J. Tallon**

United Kingdom

**Marvin Heuer**

United States of America

**Michael Roberts**

United States of America

**Neil Johannsen**

United States of America

**Nicholas Ratamess**

United States of America

**Pablo Sanchez-Soria**

Canada

**Patrick Davitt**

United States of America

**Patrick Jacobs**

United States of America

**Paul Cribb**

Australia

**Pedro Tauler**

Spain

**Peter Fitschen**

United States of America

**Rafael Longhi Sampaio de Barros**

Brazil

**Ralf Jäger**

United States of America

**Robert Olek**

Poland

**Ron Mendel**

United States of America

**Ryan Lowery**

United States of America

**Sara Campbell**

United States of America

**Sara Hayward**

United States of America

**Sean Collins**

United States of America

**Shannan Lynch**

United States of America

**Shawn Arent**

United States of America

**Shawn Wells**

United States of America

**Stephanie Dillon**

United Kingdom

**Stephanie Mihaly**

United States of America

**Stephen Bailey**

United Kingdom

**Steve Schmitz**

United States of America

**Sunita Potgieter**

South Africa

**Susan Kleiner**

United States of America

**Thomas Kjeld**

Denmark

**Tim Ziegenfuss**

United States of America

**Todd Astorino**

United States of America

**Travis Harvey**

United States of America

**Valentina Stojceska**

United Kingdom

**Victor Andrade-Souza**

Brazil

**Will Brink**

United States of America

**Xanne Janse de Jonge**

Australia

**Yang Zhang**

China

